# Improvement in light tolerance with oral *Polypodium leucotomos* extract in a patient with nonsegmental vitiligo treated with narrow-band UV-B phototherapy

**DOI:** 10.1016/j.jdcr.2024.04.035

**Published:** 2024-05-08

**Authors:** Afsheen Sharifzadeh, Heather Gochnauer, John E. Harris

**Affiliations:** a Department of Dermatology, University of Massachusetts Chan Medical School, Worcester, Massachusetts; b Department of Dermatology, New York Medical College/Metropolitan Hospital Center, New York, New York

**Keywords:** immunomodulation, photoprotection, photosensitivity, phototherapy, polypodium leucotomos, vitiligo

## Introduction

Vitiligo is a common T cell mediated autoimmune skin disease characterized by patchy depigmentation from melanocyte loss, impacting individuals' quality of life. Treatments include systemic glucocorticoids, topical corticosteroids, calcineurin inhibitors, JAK inhibitors, and narrow-band ultraviolet B (NB-UV-B).

Traditional remedies for vitiligo have informed current therapies. The revival of psoralen derivatives in treatment of vitiligo by dermatologists in the 20th century was inspired by ancient Egyptian and pre-Vedic Indian texts describing the application of psoralen-containing plant emulsions to affected skin before sunlight exposure.^1^ *Polypodium leucotomos*, a fern native to the subtropical Americas, remains a skin remedy in the Andes Mountains.^2^ Aqueous leaf extracts of this plant, or *Polypodium leucotomos* extract (PLE), are thought to confer photoprotection through antioxidative effects including quenching free radicals, lipid peroxidation, and reactive oxygen species induced by UV. Furthermore, PLE induced the shift of a type 1 to a type 2 T-cell cytokine profile.^3^ Two randomized placebo-controlled trials (RCT) have supported its synergistic effect with NB-UV-B in treatment of vitiligo in skin phototypes I to IV.^4^^,^^5^ Other studies reported systemic photoprotection at the clinical, histologic, and cellular levels when administered before UV light exposure.^2^^,^^6^ This case highlights PLE's utility in mitigating photosensitivity during NB-UV-B phototherapy, enabling treatment continuation and enhancing repigmentation in vitiligo.

## Case

MF is a healthy 54-year-old man with skin phototype III who presented for evaluation of white skin spots. He first noticed them at the age of 16 after a skateboarding accident, where he had friction injuries on both elbows and knees. He did not seek evaluation until his second SARS-CoV-2 vaccination at age 54, when he observed new spots on his forearms, calves, and dorsal feet, occasionally becoming itchy and pink.

**Fig 1:**
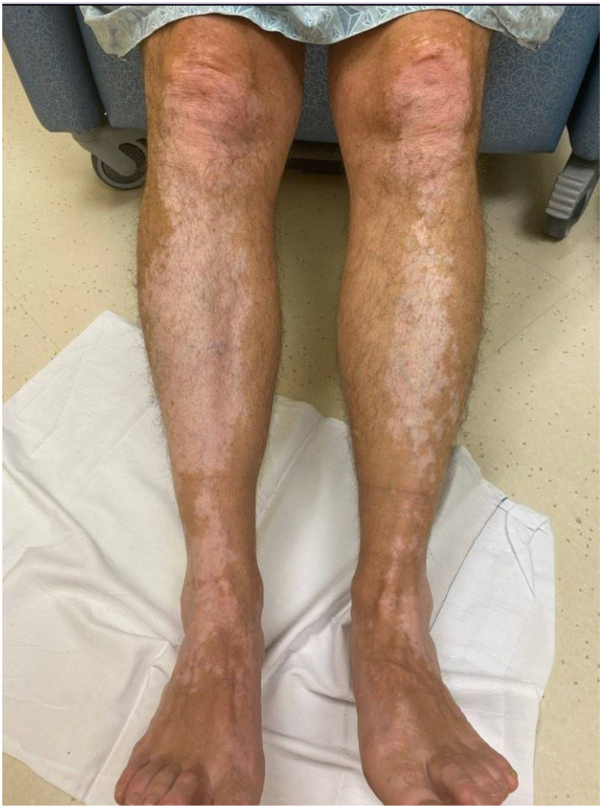
Anterior legs with well-demarcated depigmented macules and patches prior to treatment initiation.

Physical exam revealed well-demarcated, depigmented macules and patches on the bilateral elbows, forearms, dorsal hands (Fig 1), volar wrists, mid lower back, shins, calves, and dorsal feet. Lesions included confetti-like macules and evidence of koebnerization on the bilateral forearms. He was diagnosed with active vitiligo and prescribed 4 mg oral dexamethasone twice weekly for 3 months, as well as topical betamethasone 0.05% cream twice daily for 1 week, alternating with topical tacrolimus 0.1% ointment twice daily the next week. He also started in-office NB-UV-B therapy 3 times weekly.

**Fig 2:**
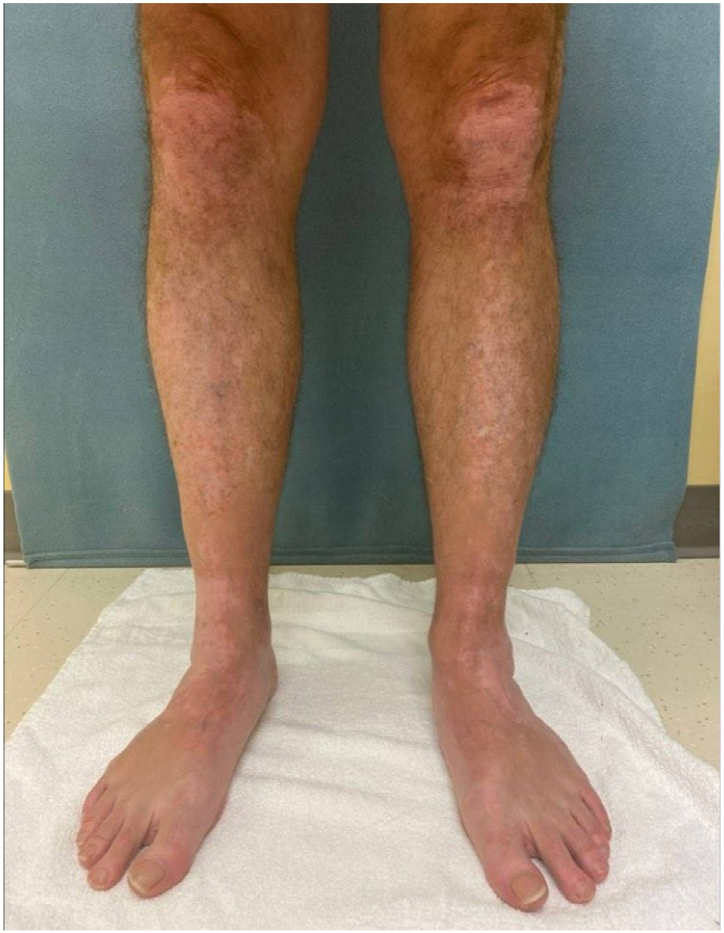
Repigmentation on anterior legs following 6 months and 12 months of treatment with *Polypodium leucotomos* extract and NB-UV-B.

Four months later, the patient reported episodes of redness and a “burning” sensation following phototherapy. At the time of evaluation, he was tolerating a NB-UV-B dose of only 375 mJ/cm^2^ over 35 seconds twice weekly due to discomfort. It was recommended that he initiate oral PLE supplementation as instructed. The patient elected to take one 240-mg capsule of oral PLE (Heliocare, Industrial Farmacéutica Cantabria)^7^ 1 to 2 hours before phototherapy sessions to reduce photosensitivity. He was directed to incrementally increase the NB-UV-B dose with initiation of PLE, aiming for mild erythema lasting 24 to 48 hours after each exposure. Within 8 weeks, he successfully increased the NB-UV-B dose to 600 mJ/cm^2^ over 2 minutes, 3 times weekly and reported resolution of burning and redness. Twelve months later, MF exhibited marked repigmentation in affected areas (Fig 2). He is being maintained on 600 mJ/cm^2^ NB-UV-B 3 times weekly and oral PLE supplementation alongside topicals, which has improved his photosensitivity and repigmentation response.

## Discussion

We report a case where oral PLE supplementation led to a successful increase in NB-UV-B dose in a vitiligo patient with photosensitivity, allowing for a 1.6-fold increase in NB-UV-B dose per session (from 375 mJ/cm^2^ to 600 mJ/cm^2^) without adverse effects, as well as increased sessions per week. The introduction of PLE supplementation coincided with vitiligo improvement, with significant repigmentation observed in all affected areas (Figs 1 and 2). With a mild reported side effect profile,^7^ our case supports previous suggestions that PLE may be underutilized in vitiligo treatment,^8^ especially in cases where photosensitivity hinders phototherapy.

PLE's effectiveness in vitiligo was first demonstrated in a double-blinded RCT comparing psoralen plus ultraviolet A combined with either PLE or a placebo in vitiligo patients. The treatment group showed a significantly higher rate of achieving over 50% skin repigmentation compared to the placebo group (*P* < .01). This response inversely correlated with CD3+ CD25+ T-cell counts.^9^ In another RCT, 50 subjects were randomly assigned to receive a placebo or 250 mg of PLE 3 times daily alongside twice-weekly NB-UV-B for 25 weeks. The treatment group achieved higher repigmentation rates (50%) in the head and neck, particularly in Fitzpatrick skin types II and III, compared to the placebo group (19%; *P* = .002).^3^ A third randomized trial involved 44 vitiligo subjects treated with either twice-weekly full-body NB-UV-B or 480 mg of PLE twice daily or NB-UV-B alone for 6 months. The combined treatment group achieved significantly higher repigmentation rates than controls (47.8% vs 22%, respectively).^5^ PLE's photoprotective effects have been explored in various clinical trials. In a study involving healthy volunteers (*n* = 9) with skin phototypes II to III, exposure to UV radiation across different wavelengths (305-400 nm) before and after taking PLE capsules (7.5 mg/kg each) led to significant reductions in mean erythema response at 24 hours (*P* < .01), decreased sunburn cells (*P* < .05), cyclobutane pyrimidine dimers (*P* < .001), proliferating epidermal cells (*P* < .001), and dermal mast cell infiltration in biopsy specimens compared to controls (*P* < .05).^6^ In a subsequent double-blinded RCT involving healthy subjects (*n* = 40), the placebo group had a 6-fold higher likelihood of experiencing at least 1 sunburn during the study compared to those taking 240 mg of PLE twice daily for 2 months (*P* = .04). Additionally, after 28 days of treatment, the treatment group exhibited a 22-fold greater odds ratio of increased UV-B minimum erythema dose compared to the placebo group (*P* = .01).^6^ A recent study in healthy volunteers (*n* = 22) with skin phototypes I to III, who underwent irradiation before and after receiving a total of 480 mg of PLE, demonstrated significant reductions in UV-B-induced clinical alterations in 17 subjects and histological changes in all subjects. The study reported an 8% reduction (*P* < .05) in average relative erythema intensity in all subjects after PLE administration, along with changes in multiple histological biomarkers associated with UV-B-induced molecular damage.^6^

We report excellent clinical improvement in photosensitivity and regimentation using 240-mg PLE 3 times weekly before NB-UV-B treatments compared to the previous lowest tested dose (240 mg twice daily for 15 days).^10^ Given evidence supporting its utility as an adjunct vitiligo treatment and its in vitro antiinflammatory properties, future studies may consider the role of PLE supplementation as systemic vitiligo maintenance therapy.

## Conflicts of interest

None disclosed.
